# Differential and spatial expression meta-analysis of genes identified in genome-wide association studies of depression

**DOI:** 10.1038/s41398-020-01127-3

**Published:** 2021-01-04

**Authors:** Wennie Wu, Derek Howard, Etienne Sibille, Leon French

**Affiliations:** 1 Institute for Medical Science, University of Toronto, Toronto, Canada; 2 Campbell Family Mental Health Research Institute, Centre for Addiction and Mental Health, Toronto, Canada; 3 Krembil Centre for Neuroinformatics, Centre for Addiction and Mental Health, Toronto, ON Canada; 4 Department of Psychiatry, Faculty of Medicine, University of Toronto, Toronto, ON Canada; 5 Department of Pharmacology and Toxicology, University of Toronto, Toronto, ON Canada

**Keywords:** Depression, Biomarkers, Genomics

## Abstract

Major depressive disorder (MDD) is the most prevalent psychiatric disorder worldwide and affects individuals of all ages. It causes significant psychosocial impairments and is a major cause of disability. A recent consortium study identified 102 genetic variants and 269 genes associated with depression. To provide targets for future depression research, we prioritized these recently identified genes using expression data. We examined the differential expression of these genes in three studies that profiled gene expression of MDD cases and controls across multiple brain regions. In addition, we integrated anatomical expression information to determine which brain regions and transcriptomic cell types highly express the candidate genes. We highlight 12 of the 269 genes with the most consistent differential expression: *MANEA*, *UBE2M*, *CKB*, *ITPR3*, *SPRY2*, *SAMD5*, *TMEM106B*, *ZC3H7B*, *LST1*, *ASXL3, ZNF184* and *HSPA1A*. The majority of these top genes were found to have sex-specific differential expression. We place greater emphasis on *ZNF184* as it is the top gene in a more conservative analysis of the 269. Specifically, the differential expression of *ZNF184* was strongest in subcortical regions in males and females. Anatomically, our results suggest the importance of the dorsal lateral geniculate nucleus, cholinergic, monoaminergic and enteric neurons. These findings provide a guide for targeted experiments to advance our understanding of the genetic underpinnings of depression.

## Introduction

Major depressive disorder (MDD) is a leading cause of disability and a large contributor to morbidity and mortality, with an estimated annual prevalence affecting over 4.4% of the world’s population^1^. MDD is clinically diagnosed and characterized by prolonged periods of low mood or anhedonia in addition to physical and cognitive symptoms making it a complex and heterogeneous disorder^2^. The heritability of MDD, estimated through twin studies, is 31–42%, which is considered modest^3,4^. Genome-wide association studies (GWAS) are performed to identify the common variants that increase the risk of a genetic disease. However, due to the complex nature of MDD, initial GWAS were unable to identify reproducible genetic loci, potentially suggesting that many genetic factors of small-effect contribute to the overall disease manifestation^5–8^. Moreover, genes and pathways affected differ between males and females^9–14^, which may explain some variability observed in depression phenotypes. To identify genetic variants of smaller effect, a consortium effort acquired higher power by profiling larger sample sizes. This increase was achieved by including individuals that displayed broader phenotypes of depression. Cohorts that include individuals with MDD and broader depression phenotypes were defined in the recent GWAS as depression^15^.

**Fig. 1:**
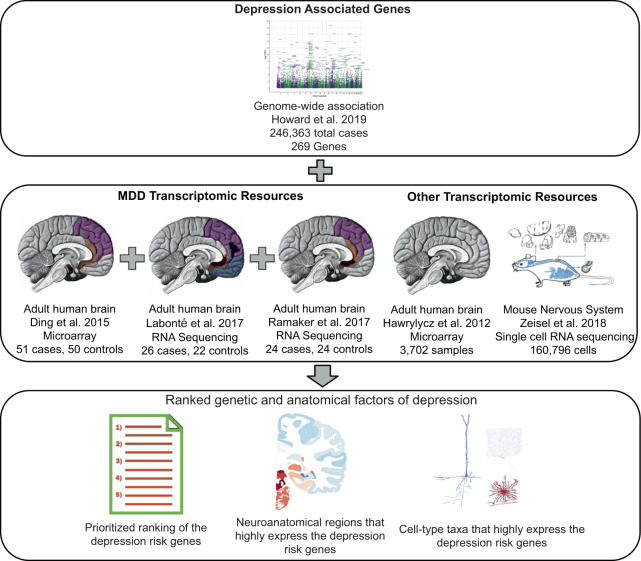
Overview of this study. The 269 genes implicated with depression (top) are characterized by several transcriptomic studies (middle). Highlighted are the different brain regions sampled within each study (middle) that will help prioritize the genes (bottom). Other transcriptomic resources that were used (middle) will identify anatomical targets associated with the disease (bottom). Images are from the cited publications, Dr. David M Howard, and Wikimedia Commons (Gray’s Anatomy by Henry Vandyke Carter).

Howard et al. conducted the largest GWAS of depression to date (total *n* = 807,553) by meta-analyzing data from three previous studies of depression: Hyde et al.^16^, Howard et al.^17^ and Wray et al.^18^. This large sample size resulted in the identification of 102 independent variants and 269 genes associated with depression^15^. Additionally, they found that the genes near the identified variants were expressed at higher levels in the frontal cortex and within neuronal cell types of the brain through a partitioned heritability approach using transcriptomic resources. Their results provided significant insights into the etiology of depression. However, few of the 269 genes have been studied in the context of the disorder. Furthermore, their enrichment results were based on 13 brain regions and three brain cell types. To provide additional context, we examined these genes in studies that have profiled gene expression in postmortem brain samples of MDD cases. We hypothesized that genes with greater genetic associations would be differentially expressed in these transcriptomic studies of MDD. We performed a differential expression meta-analysis to prioritize the 269 genes and tested for evidence of opposing molecular signals between males and females. In addition, we used large transcriptomic atlases to obtain a finer perspective on the specific anatomy associated with the genetic findings. Our hypothesis for this analysis was that the prefrontal cortex and neuronal cell types are more enriched for the expression of the 269 genes. Figure 1 provides an overview of these analyses. Ultimately, we sought to provide guidance for future studies of depression by narrowing genetic and anatomical targets.

## Methods

### Depression GWAS data

The latest GWAS of depression included 246,363 cases and identified 102 genetic variants. The included cohorts measured a broad range of phenotypes that included nerves, tension, self-reported depression and impairment, and clinically diagnosed depression. For example, the UK Biobank cohort included broad depression phenotypes and the 23andMe cohort assessed phenotypic status based on the responses provided in online surveys and that self-reported being diagnosed with depression by a professional. As the majority of the included participants did not have MDD, this was defined as a study of depression^15^. To summarize the variant to gene associations, Howard et al. used the MAGMA (Multi-marker Analysis of GenoMic Annotation) tool^19^. Genome-wide, MAGMA aggregated the genetic variants associated with depression to reveal the 269 of 17,842 tested genes that passed the multiple test correction threshold. Our analyses focused on these 269 depression risk genes.

### MDD transcriptomic studies

MDD transcriptomic studies were selected based on the following criteria: transcriptomic profiles were obtained from human postmortem brain tissues, cases were diagnosed with MDD, results of the study included data from each sex, and the study was published within the past five years. A summary of the transcriptomic datasets used in our meta-analysis is presented in Table 1. The cases in each dataset were diagnosed with MDD through psychological autopsies that included interviews with family or individuals best-acquainted with the deceased. More information is outlined in the respective studies^13,20,21^.

**Table 1:** Characteristics of the MDD transcriptomic datasets.

Reference	Assay type	Brain regions	Sample size MDD: CTRL Female %	
Ding et al.^20^	Microarray	1. Dorsolateral prefrontal cortex^a^ 2. Subgenual anterior cingulate cortex^a^ 3. Rostral amygdala	51: 50	49
Labonté et al.^13^	RNA Sequencing	1. Orbitofrontal cortex (BA11) 2. Dorsolateral prefrontal cortex (BA8/9)^a^ 3. Subgenual prefrontal cortex (BA25)^a^ 4. Anterior Insula 5. Ventral Subiculum 6. Nucleus Accumbens^b^	26: 22	40
Ramaker et al.^21^	RNA Sequencing	1. Dorsolateral prefrontal cortex^a^ 2. Anterior cingulate gyrus^a^ 3. Nucleus accumbens^b^	24: 24	25

*MDD* major depressive disorder, *CTRL* control.

^a^Brain regions assayed in all three studies.

^b^Brain regions assayed in two studies.

### Ding et al. transcriptomic analyses

Using microarray expression profiling, Ding et al. analyzed 101 human postmortem subjects (Table 1)^20^. Eight studies were conducted between the two sexes in three corticolimbic regions: four studies were performed in the subgenual anterior cingulate cortex, two in the amygdala and two in the dorsolateral prefrontal cortex. Initially, 16,689 unique genes were assayed across all studies but were further reduced. Firstly, genes were ranked based on expression level, and the lowest 20% of genes were considered non-expressed and filtered out. Then, genes were ranked based on the variation of expression and the lowest 20% were filtered out. This left Ding and colleagues with 10,680 genes. For each gene, they provided eight single-study *p*-values and effect sizes (one from each sex-specific study) that we used in our analyses. These statistics were calculated with a random intercept model combined with Bayesian information criterion for parameter selection by Ding and colleagues^20^.

### Labonté et al. transcriptomic analyses

Labonté et al. examined gene expression profiles of 48 human postmortem brains (Table 1) and reported sex-specific transcriptional signatures of MDD using RNA sequencing. They sampled from six corticolimbic structures: the subgenual prefrontal cortex (BA25), orbitofrontal cortex (BA11), dorsolateral prefrontal cortex (BA8/9), anterior insula, nucleus accumbens and ventral subiculum^13^. Genome-wide results were provided by Labonté and colleagues and are available in our GitHub repository (24,943 genes). Of those genes, 20,386 had *p*-values, and the associated log fold change values for both sexes in each brain region (12 *p*-values per gene), which were used in our analyses.

### Ramaker et al. transcriptomic analyses

Samples from the anterior cingulate cortex, dorsolateral prefrontal cortex and the nucleus accumbens were profiled by Ramaker et al. using RNA sequencing. We used data from the controls and those with MDD for a total of 48 subjects^21^. We re-processed the metadata and raw count files obtained from GSE80655 using the BioJupies R package referencing the methods in their paper^22^. For the differential expression analysis, we included the same covariates as outlined in their paper: age, brain pH (pH), disorder (MDD), postmortem interval (PMI) and percentage of reads uniquely aligned (PRUA). Unlike the other two transcriptomic studies, Ramaker et al. did not include sex as a covariate. The normalized data was transformed to log2-counts per million using the limma’s R package voom function to be linearly fitted with the full design model previously mentioned using limma’s lmFit function^23–25^. The differentially expressed data was then calculated from the linear fit model using limma’s eBayes function^24^. This resulted in 35,238 genes with the associated *p*- and *t*-values for each brain region for both sexes for downstream meta-analyses.

### Differential expression statistics

We integrated differential expression statistics at the level of genes and found that most of the 269 GWAS identified genes were assayed in at least two transcriptomic studies. The Ding et al. dataset provided differential expression statistics for 155 of the 269 depression risk genes. Of the 114 genes without data, 68.4% were filtered out due to the study’s filtering criteria, and the remaining 31.6% were uncharacterized in this study. Labonté et al. had complete differential expression data for 243 of the 269 genes. For the 26 missing genes, seven genes did not have *p*-values for both sexes across their sampled brain regions and were filtered out from our analysis. The remaining 19 genes were found to be assayed in the dataset (GSE102556), but appear to have been filtered out by the analysis pipeline of Labonté and colleagues. However, Ding et al. also filtered out 14 of the 19 genes suggesting they had low expression levels and variance. For the Ramaker et al. dataset, we re-analyzed the corresponding dataset (GSE80655), resulting in differential expression statistics for all 269 genes. Overall, differential expression statistics from all three transcriptomic studies were available for 153 of the 269 depression risk genes.

### Meta-analysis

We performed study-specific meta-analyses that combined across sexes and brain regions in a single study and broader meta-analyses that joined results across studies. These meta-analyses followed one of five criteria that differ in the number of brain regions or sexes across the transcriptomic studies. For instance, the full analysis included data from all brain regions and both sexes. We also separated female from male data across all brain regions to identify sex-specific effects. The expression patterns across the cortex are relatively stable compared to the larger expression differences found across the subcortex^26^. To limit regional variability, we performed separate analyses that were restricted to cortical and subcortical samples. Select criteria were applied in our three developed models to highlight candidate genes associated with the different objectives of the models, which are further described in the sections below.

Our meta-analysis methods differed depending on the model under analysis, but all followed the same general process. First, genes were prioritized in association with MDD by performing a meta-analysis in each transcriptomic dataset. For each study, we combined the one-sided *p*-values across the desired sex and brain regions for each gene in both directions of expression change using Fisher’s combined probability test^27^. The direction with the more significant *p*-value was used to calculate the two-sided study-specific meta *p*-value and meta direction. To aggregate the three study-specific meta-analyses into one across-study meta-analysis, the one-sided study-specific *p*-values for each gene were combined using Fisher’s method in each direction^27^. The across-study meta direction and meta *p*-values for each gene were calculated as described above. The Bonferroni method was used to correct for multiple testing.

### First model

Our first model was the simplest, where the objective was to identify the genes that were consistently differentially expressed across the three transcriptomic datasets under the five meta-analysis criteria.

### Sex-interaction model

Opposing sex-specific patterns have been previously reported in transcriptomic studies of MDD^13,14^. This model’s objective was to test for genes with opposing transcriptional differences between male and female cases of MDD. To do this, we inverted each gene’s direction of differential expression (multiplied by −1) for males before performing our study-specific meta-analyses. Genes were prioritized under our full, cortical and subcortical criteria.

### Genome-wide ranking model

This model was designed to equally weight the per-gene statistics of each study, providing a relative assessment of the gene’s significance compared to the rest of the genome. This model was applied to the results of the eight study-specific meta-analyses.

This model uses genome-wide study-specific meta-analysis results from the other two models. Howard et al. identified the 269 depression risk genes by testing 17,842 genes using MAGMA^19^. Therefore, we filtered our study-specific results to select for those included in the 17,842 gene set. An additional step was then taken compared to the other models to convert the absolute *p*-value to a genome-wide relative statistic. In every study-specific meta-analysis result from each model, we calculated the proportion of genes in the genome with a smaller study-specific meta *p*-value than the current gene under observation in both directions (higher and lower expression in cases). For example, a gene with a study-specific *p*-value of 1.56 × 10^−6^ that ranks 100th genome-wide, would be assigned an empirical *p*-value of 100/17842 = 0.0056. This procedure is applied for all three studies, providing a similar uniform distribution of *p*-values across the genome. Then, as with the other models, these *p*-values for each gene and direction were combined across studies using Fisher’s method as described above.

### Genetic and transcriptomic associations

We investigated the degree of association between our across-study meta-analyses results and the gene-based MAGMA statistics for the 269 genes using Spearman correlation. We also tested if our across-study meta-analyses statistics significantly differed for the 269 genes compared to the 17,573 genes that were not associated with depression using the Wilcoxon rank-sum test.

### Neuroanatomical expression enrichment

The Allen Human Brain Atlas, a comprehensive transcriptomic atlas of the human brain, was used to characterize neuroanatomical expression patterns^28^. This atlas mapped the human brain’s transcriptomic architecture from six healthy adults of five males and one female (ages 24–57). This atlas contains 3702 expression profiles of 232 distinct brain regions.

Using this atlas, we created a maximum expression map that assigns the brain region that maximally expresses each of the 269 depression risk genes. We used the probe-to-gene mappings generated by the Re-Annotator software^29^. Some regions were profiled from a single donor resulting in some donor-specific bias. To reduce this bias, we filtered the brain regions that included expression data from at least four donors leaving 190 brain regions. Probe level expression values were averaged for each gene transcript across the donors in the 190 brain regions. We then filtered for the region with the greatest expression for each gene, creating our maximum expression gene-to-region mapping. We used the hypergeometric test to identify if any region was significantly enriched for maximal expression.

### Cell-type taxon expression enrichment

Zeisel et al. used single-cell RNA sequencing to characterize the transcriptomic cell types within the mouse nervous system^30^. They obtained the transcriptome of 509,876 cells, which was reduced to 160,796 cells after assessing quality. These remaining cells formed 265 transcriptomic cell-type clusters, which were broadly grouped into 39 distinct cell-type taxa across the central and peripheral nervous systems.

We referenced these results to map the 269 genes to the cell-type taxon that most highly expresses it. We downloaded the study’s publicly available expression matrix (level 6 taxon level 4 aggregated all cell types) loom file found at http://mousebrain.org/loomfiles_level_L6.html. This expression matrix provides the average molecule counts for each cell-type taxon. The taxon that displayed the highest expression for each gene was selected to create our maximum expression map. The R homologene package was used to map the 269 genes to orthologous mouse genes^31^. The hypergeometric test was used to identify taxa enriched for maximal expression of the depression risk genes, and their z-scores across the 39 taxa were calculated.

## Results

We prioritized the 269 depression risk genes identified in the most recent GWAS of depression. Differential expression statistics were obtained from three transcriptomic studies that examined expression in a total of 197 postmortem brains (101 MDD cases and 96 control subjects, Table 1). These studies focused on the cerebral cortex by sampling from the orbitofrontal, dorsolateral prefrontal, insular, and anterior cingulate cortices. Subcortical samples from the rostral amygdala, nucleus accumbens and the ventral subiculum were also transcriptomically profiled. Of these, the dorsolateral prefrontal and anterior cingulate were profiled in all three studies.

### Full across-study meta-analysis

**Fig. 2:**
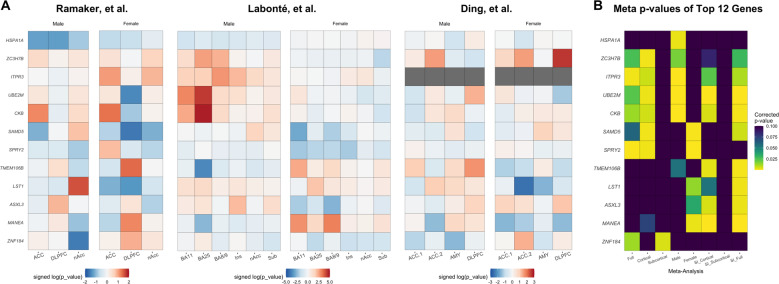
Heatmap visualizations of differential expression results. **a** Study-specific direction signed log(*p*-values) for the top 12 genes separated by sex and region. Cell colours range from blue to red, which represents lower and higher expression in cases compared to controls, respectively. Colour intensity represents the degree of differential expression. Missing values are marked in gray. **b** Corrected meta *p*-values for the same genes across the 8 across-study meta-analyses. Cell colours range from low (yellow) to high (purple) corrected *p*-values in each meta-analysis. *ACC* anterior cingulate cortex (two studies)*, DLPFC* dorsal lateral prefrontal cortex*, nAcc* nucleus accumbens*, Ins* anterior insula*, Sub* subiculum*, AMY* amygdala, SI sex interaction.

Beginning with the broadest prioritization perspective, we were interested in identifying the depression risk genes that were most consistently differentially expressed across all brain regions and both sexes. Our full across-study meta-analysis was a result of combining 26 *p*-values across the study-specific meta-analyses. In this analysis, two genes were differentially expressed: *SPRY2* (*p*_Bonf_ < 0.00347) with lower levels of expression and *ITPR3* (*p*_Bonf_ < 0.0161) with higher levels of expression in cases (Supplement Data Table S1, Fig. 2). Visualization of the differential expression statistics for *SPRY2* showed overall lower expression in MDD cases, while *ITPR3* was more variable across the two datasets with available data (Fig. 2). All across-study meta-analysis results are also available online as interactive spreadsheets (see Data availability).

### Sex-specific across-study meta-analysis

Evidence of gender differences has been previously shown in MDD^13,14,32^. Therefore, we performed a stratified analysis to test if any depression risk genes were differentially expressed in a sex-specific manner. When restricted to male data, four genes were statistically significant: *UBE2M*, *CKB*, *ITPR3*, all with higher expression and *HSPA1A* (all *p*_Bonf_ < 0.0249) had lower expression in MDD cases (Supplement Data Table S2, Fig. 2). For females, three genes were differentially expressed: *SPRY2* and *SAMD5* had lower levels of expression and *MANEA* (all *p*_Bonf_ < 0.0257) displayed higher levels of expression in MDD cases (Supplement Data Table S3, Fig. 2).

### Cortical and subcortical across-study meta-analysis

Cortical structures are common targets of depression research, and expression patterns across the cerebral cortex are more consistent than subcortical tissues^26,33–38^. Therefore, we restricted our analysis to cortical brain regions in both sexes by combining 18 region and sex-specific analyses. This highlighted four statistically significant genes: *SAMD5*, with lower levels of expression, *ZC3H7B* with higher levels of expression, *SPRY2* with lower expression and *UBE2M* with higher levels of expression in MDD cases (all *p*_Bonf_ < 0.0202). *ZC3H7B* was the only gene that was not identified in the above analyses, suggesting a stronger cortex-specific signal for this gene (Supplement Data Table S4, Fig. 2). The other three remaining genes were previously identified in the above meta-analyses.

We additionally performed a subcortical across-study analysis that combined eight region and sex-specific analyses. This analysis highlighted one gene *ZNF184* (*p*_Bonf_ < 0.0457), suggesting a specific subcortex signal with overall lower expression in MDD cases (Supplement Data Table S5, Fig. 2).

The cortical meta-analyses, which resulted in four differentially expressed genes in comparison to the single gene identified in the subcortical data suggest a stronger cortical signal. However, fewer regions were included in the subcortical analysis, reducing the power to detect consistent differential expression. To test this effect, we reduced the cortical meta-analyses to the same number of region and sex-specific analyses used in the subcortical analyses (eight). Of the 36 possible cortical combinations with matching power, 32 had no, or a single differentially expressed gene. This suggests our limited findings in the subcortical meta-analysis is due to less sampling of the subcortex, and expression differences are not enriched in cortical regions.

### Sex-interaction across-study analyses

Previous analyses using the Ding and Labonté datasets have found that differentially expressed genes showed inverse expression differences between male and female MDD cases^13,14^. To determine if this applied to the 269 genes, we tested for opposing transcriptional changes. Using data from all assayed brain regions, we found *MANEA*, *UBE2M*, *TMEM106B*, *CKB*, *LST1* and *ASXL3* were differentially expressed in opposing directions between sexes (all *p*_Bonf_ < 0.0235, Supplement Data Table S6, Fig. 2). When we restrict the interaction analysis to cortical samples, the same genes were identified except *LST1* and *ASXL3* (Supplement Data Table S7, Fig. 2), and when restricted to subcortical brain regions, no genes were differentially expressed (Supplementary Data Table S8, Fig. 2). Given this increased number of hits, we additionally tested if our meta-p values are lower across all 269 genes and found they are not (paired Wilcoxon rank-sum test, *p* = 0.24). While these results are limited, the increased number of hits from this model provides some support for previous findings of opposing gene expression signatures of MDD between males and females.

### Genome-wide ranking analyses

The Labonté dataset had greater influence in our across-study meta-analysis results. Specifically, *ITPR3* and *SPRY2* that were found in our full analysis from the first model were only significant in the full Labonté-specific meta-analysis. The full Labonté-specific meta-analysis also had the lowest *p*-values across the 269 genes (Labonté =1.56 × 10^−6^; Ding = 3.3 × 10^−5^; Ramaker = 3.38 × 10^−4^). Labonté et al. assayed more regions, which possibly amplified donor-dependent signals when *p*-values were combined. Therefore, to equalize the contributions of each study, we derived normalized ranks for each gene, relative to the rest of the genome (see “Methods”). Across the eight genome-wide meta-analyses, top genes were *ZNF184* from the subcortical analysis of the first model (empirical *p*_Bonf_ = 0.156), *PSORS1C1* from the cortical analysis in the sex-interaction model (empirical *p*_Bonf_ = 0.184) and *HSPA1A* in the male analysis from the first model (empirical *p*_Bonf_ = 0.262) (Supplement Data Table S9–S11). The top genes from the remaining five meta-analyses had an empirical *p*_Bonf_ of 1 (Supplement Data Table S12–S16). Although there is a significant loss of power, when the 269 genes are analyzed relative to the remainder of the genome *ZNF184* shows the most consistent differential expression when studies are equally weighted.

### Broad associations between genetic and transcriptomic results

Beyond individual gene tests, we assessed broader relationships between the genetic and differential expression results. In our 16 across-study meta-analyses, there was no correlation between the genetic and differential expression statistics (|*r* | < 0.04, *p* > 0.0598) and no significant difference between the statistics for the 269 genes and the 17,573 tested genes not associated with depression (Wilcoxon rank-sum test). Overall, a broad association between the genetic and gene expression signals was not observed.

### Neuroanatomical expression enrichment

To provide a spatial perspective, we created a maximal expression map that links each depression risk gene to the brain region that most highly expresses it. To reduce donor-specific sampling biases from the Allen Human Brain Atlas, we examined 190 regions that were all assayed from at least four donors. With the exception of *C7orf72*, the remaining 268 genes were profiled in this Atlas. Seventy-nine brain regions maximally expressed at least one of the 268 genes. Given this large number of regions, we tested if specific brain regions were significantly enriched for maximal expression of the 268 genes than expected by chance (Supplementary Data Table S17). The midbrain raphe nuclei had the strongest enrichment for maximal expression (*p*_Bonf_ = 0.021). The six genes that were maximally expressed in this region are all members of the protocadherin alpha family (*PCDHA1, PCDHA2, PCDHA3, PCDHA4*, *PCDHA5, PCDHA7*). These genes form a cluster on chromosome 5 and have very similar sequences that can cause a single microarray probe to match several protocadherin genes^39^. This was reflected in our results where three genes (*PCDHA2, PCDHA4*, *PCDHA7)* were mapped to the same probes. After grouping these protocadherin genes together, enrichment of the midbrain raphe nuclei was no longer statistically significant; and the top brain region was replaced by the dorsal lateral geniculate nucleus of the thalamus (15 genes maximally expressed, *p*_Bonf_ = 0.0806). The map showed the central glial substance maximally expressed the most genes (26 genes) but was not statistically significantly enriched (*p*_Bonf_ = 1) (Supplementary Data Table S17). The combined corticolimbic structures maximally expressed 36 of the 268 genes indicating that the majority of depression associated genes are highly expressed in other brain regions. Therefore, a diverse set of regions are highly enriched for the depression risk genes.

### Cell-type taxon expression enrichment

**Fig. 3:**
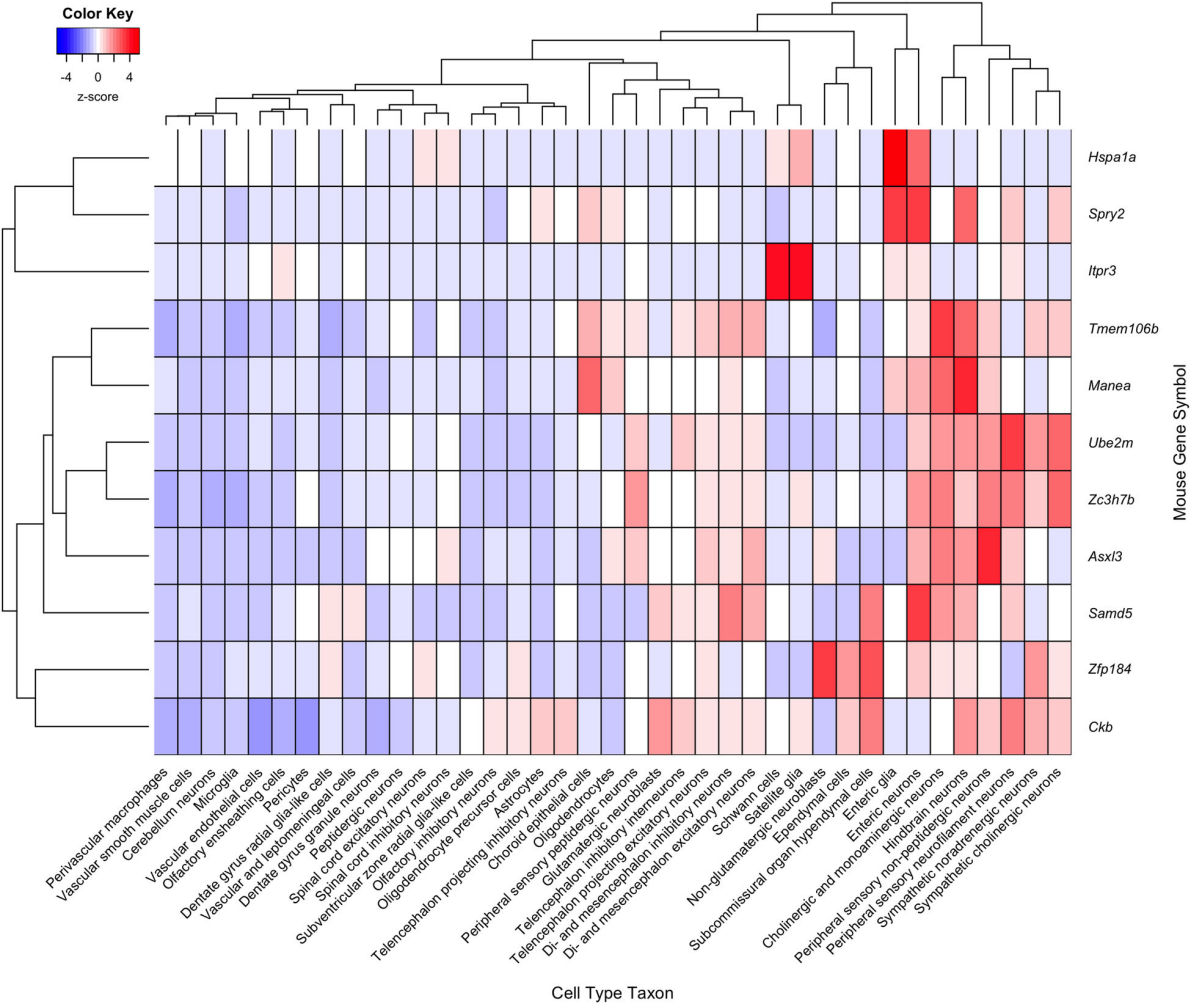
Expression heatmap for the top differentially expressed genes across the 39 mouse cell-type taxa. Cell colours range from blue to red, which represent depleted and specific expression, respectively. *LST1* is not shown because it lacks a homologous mouse gene in the Homologene database.

We next sought to identify cellular populations enriched for expression of the 269 depression risk genes. We created a maximum expression map of the cell-type taxon that most highly expresses each gene. Transcriptomic cell types were obtained from a clustering of cells from the mouse nervous system^30^. This maximum expression map summarizes the cell-type taxon maximally enriched for each depression risk gene. Of the 269 depression risk genes, 240 had orthologous mouse genes with expression data available. Of the 39 transcriptomic cell-type taxons, 34 had maximal expression of at least one of the risk genes. Two transcriptomic cell types were enriched for maximal expression: cholinergic and monoaminergic neurons (*p*_Bonf_ = 2.26 × 10^−5^) and enteric neurons (*p*_Bonf_ = 0.00893) (Supplementary Data Table S18). In Fig. 3, expression across all 39 cell-type taxa is presented for the top differentially expressed genes. This marks the diffuse pattern of *Ckb* (max z-score = 2.2) and specific expression of *Hspa1a* and *Spry2* in enteric cells (max z-score > 3.4). The enteric neuron taxon included neuronal cell clusters annotated as nitrergic and cholinergic^30^. Deeper analysis using the more granular 265 transcriptomic cell-type clusters indicated that enteric cholinergic neurons had a greater enrichment than nitrergic enteric neurons (Supplementary Data Table S19). The cholinergic and monoaminergic neuron taxon contains clusters that express various neurotransmitters and are localized to the mid- and hindbrain^30^. Within this taxon, greatest enrichment was observed in the cluster named ‘afferent nuclei of cranial nerves VI–XII’ followed by clusters of cholinergic and serotonergic neurons (Supplementary Data Table S19). In this mouse dataset, we again observe a diverse anatomical pattern for the 269 genes.

### Predictability of Gene Expression

To assess how specific the differential expression signals are to depression, we examined the depression associated genes in the context of a large differential expression meta-analysis^40^. This meta-analysis calculated the prior probabilities for a list of genes. The higher the probability, the more likely that gene will be differentially expressed for many case-control disease studies. We included these empirical prior probabilities for the 269 genes in our result tables (Supplement Data Tables S1-S16).

For our top 12 genes, data for *UBE2M* was not available, and the remaining genes had empirical prior probabilities above 0.732 except for *ZNF184* (0.368) and *ZC3H7B* (0.183). These results suggest that on an individual gene basis, differential expression of *ZC3H7B* and *ZNF184* are specific to depression while the other nine genes may be perturbed by generic processes.

### Interactive online spreadsheet

We provide all our tables as interactive online spreadsheets to promote collaborative information sharing for these 269 genes. Across-study meta-analysis results are available online as interactive spreadsheets (see Data availability). Comments are enabled, and edit access can be requested to add information as we learn more about these candidate causal genes.

## Discussion

We prioritized the genes identified in the largest genetic study of depression to date by incorporating differential expression data from 197 individuals across seven unique brain regions related to reward, attention and emotion processing. We highlight 12 genes with the most consistent differential expression. Referencing transcriptomic atlases, we find that these genes are broadly expressed with some enrichment in the dorsal lateral geniculate nucleus, cholinergic, monoaminergic, and enteric neurons. Our study highlights relevant pathogenic tissues and candidate causal genes to guide future studies of depression risk factors.

Dysfunction in prefrontal cortical circuits is commonly implicated in depression pathogenesis^15,18,41–43^. Furthermore, these regions primarily play a role in executive functions and emotion regulation, which are often impaired in depression^33–38,44,45^. Prior focus on the frontal cortex may have indirectly inflated its relevance to the disorder. For example, in schizophrenia, a larger number of dorsolateral prefrontal cortex associations from a transcriptome imputation analysis was driven by tissue sample size rather than the relevance of the region^7^. Howard et al. found that genes harbouring the genetic variants have specific expression enrichment in the healthy prefrontal cortex. However, in our analysis, the dorsal lateral geniculate nucleus of the thalamus was most enriched for the depression risk genes. This region that relays visual information most highly expresses *CKB*. In addition, *MANEA*, another top hit, is highly expressed in the nearby dorsolateral thalamus. Past studies have explored the association between vision impairment and depression^46–51^. Research has also identified possible sex differences related to visual perception^52^. Our lack of enrichment in the frontal cortex may be a result of our focus on the 269 genes and the finer anatomical resolution of our analyses. We suspect that experiments targeting these specific regions and genes may provide deeper insight into depression.

We provide evidence that neurons are enriched for the expression of candidate depression risk genes than expected by chance. Our findings highlighted enteric neurons, supporting previous associations between the gut microbiome and mental health^53^. Furthermore, integration of the depression GWAS results and transcriptomic data from brain and non-brain tissues found enrichment in the colon^7^. Future research should continue to explore the potential associations between the enteric nervous system and mood disorders.

Broadly, we observed no correlation between differential expression in MDD and the degree of genetic association. Similar findings were also reported in a meta-analysis of autism spectrum disorder^54^. Past consortium analysis identified 108 loci associated with schizophrenia, comparable to the 102 loci associated with depression^55^. In a transcriptomic study of schizophrenia, two genes harbouring the 108 loci were differentially expressed in the prefrontal cortex^56^. In addition, epigenetic risk scores for depression are largely independent of the polygenic genetic risk scores^57,58^. Given these previous findings of weak relationships between differential expression, methylation, and genetic hits, our number of highlighted genes is not unexpected.

Mirroring our *CKB* results, creatine studies have also found sex-specific signals in the context of depression. Recently, *CKB* was also differentially expressed in a single-nucleus study of the prefrontal cortex in MDD^59^. Creatine kinase isoenzymes, including CKB, which is specific to the brain, converts creatine to phosphocreatine to efficiently meet energy demands^60^. In rodents, creatine kinase isoenzymes are sexually dimorphic with higher activity in males than females^61^. The Human Protein Atlas indicated *CKB* is expressed at higher levels in male versus female tissues^62^. In MDD studies, increased creatine levels heightened depressive symptoms in male rats while females displayed antidepressant-like effects^63^. Phosphocreatine levels and depression scores were negatively correlated in the frontal lobe in adolescent females with treatment-resistant MDD^64^. Recently, a negative relationship between dietary creatine consumption and depression was found in an American sample of 22 692 adults^65^. When stratified by sex, this effect was only statistically significant in females. In support of past studies, our findings warrant further investigation of *CKB* activity and creatine concentrations in the context of depression.

There is a genetic correlation between depression and obesity, and shared genetic factors include Sprouty RTK Signaling Antagonist 2 (*SPRY2*)^15,18,66^. *SPRY2* was significantly associated with body fat percentage and type 2 diabetes mellitus in large genetic studies^67–69^. A knockout analysis of *SPRY2* found a significant increase in glucose uptake and lipid droplet accumulation in an in vitro model of human hepatocyte cells^70^. This suggests that decreased expression of *SPRY2* in human hepatocytes contributes to the pathogenesis of obesity and type 2 diabetes. MDD severity in females was correlated with various measures of obesity (BMI, total body fat and visceral fat mass)^71^. Our results reflect that *SPRY2* is more female-specific, with overall decreased levels of expression in cases. Additionally, *SPRY2* is most highly expressed in enteric neurons suggesting an association with the gut-brain-axis. Further genetic studies may reveal the role of *SPRY2* in both depression and obesity, particularly in females.

*UBE2M* has been associated with various cancers^72–74^, and dermatomyositis^75^. These illnesses predominantly affect males and commonly have overactivation of *UBE2M* that generally results in poorer survival^72–75^. Similarly, we show that *UBE2M* is a more male-specific gene with greater expression in MDD cases. Additionally, *UBE2M* is most highly expressed in peripheral sensory neurons, which are also affected in some cases of dermatomyositis^76–82^. Further studies are needed to better understand this gene in the context of both dermatomyositis and depression.

Although *ITPR3* was filtered from the Ding et al. study, it remained highly prioritized with higher expression in cases, particularly males. This gene encodes a receptor protein that mediates the intracellular release of calcium^83^. In our analysis, *ITPR3* was most highly expressed in the supraoptic nucleus of the hypothalamus. This region produces vasopressin, an antidiuretic hormone^84,85^. Past studies found that MDD cases have increased vasopressin plasma concentrations, which were also found to be positively correlated with psychomotor retardation^86–88^. Inositol and its supplementation have been studied in the context of depression with mixed results (reviewed in ref. ^89^). Additional studies are needed to assess the interrelationship between *ITPR3*, vasopressin, inositol, calcium and depression.

The limitations of this study are consistent with those inherent in most postmortem brain gene expression studies and must be considered when interpreting our results. By combining datasets, we sought to alleviate challenges associated with low sample size and choice of brain regions assayed. Signals of RNA quality, postmortem interval, and patient drug use may still be present despite efforts to control for these factors. Also, the differential expression signature of depression in the brain appears to be weak. When the individual publications are considered separately, two of the three did not identify differentially expressed genes after multiple test correction with Ding and colleagues identifying only nine. When examining each of the 26 studies in isolation, only two genes survive correction for 269 tests (*CKB* and *UBE2M*). Another limitation is that our use of Fisher’s method assumes the independence of gene expression profiles from different regions of the same donor. As a result, the correlation between expression profiles from different regions of the same donor will probably boost signals repeated across brain regions. We performed ranked and cortical/subcortical analyses to address this, but note our analyses are biased towards expression differences that are consistent across brain regions. We also note that not all of the 269 genes had data from all three transcriptomic studies. Furthermore, the cell-type results are based on mouse rather than human data, which may not accurately translate to humans. This species difference also resulted in missing cell-type taxon assignment for some of the 269 depression risk genes without a mouse homolog. While the neuroanatomical enrichment analyses were performed on pathologically normal brains, we hope that our results will help target future cell and region-specific studies.

## Conclusion

We prioritized the 269 GWAS depression risk genes and highlighted 12 that were consistently differentially expressed across three transcriptomic studies of MDD: *MANEA*, *UBE2M*, *CKB*, *ITPR3*, *SPRY2*, *SAMD5*, *TMEM106B*, *ZC3H7B*, *LST1*, *ASXL3, ZNF184* and *HSPA1A*. We provide evidence of greater influence from sex compared to the brain region profiled. Our results revealed the depression risk genes are maximally expressed in various brain regions but highlight the dorsal lateral geniculate nucleus of the thalamus. In the mouse nervous system, cholinergic, monoaminergic, and enteric neurons highly express the candidate genes. Characterization of where these genes are most expressed revealed a diversity of regions, supporting depression’s heterogeneous nature. Overall, our results contribute important information to guide future studies and advance our understanding of the etiology of depression.

## Supplementary information

All supplement tables combined

## Data Availability

First and sex-interaction model results (Supplement data table S1-8): https://docs.google.com/spreadsheets/d/1L2viS9-TrikGeGulVTBcAUm031VEjqZbW4IQRLWKQ6c/edit?usp=sharing, Genome-wide ranking model results (Supplement data table S9-16): https://docs.google.com/spreadsheets/d/1xjGj5Cx4K0PlKOjMlMXT67-19rYBA18bJUWkxU_YtEk/edit?usp=sharing.

## Code availability

All data and results are publicly available at https://github.com/computationalneurobiology/MDD269Meta.

## References

[1] World Health Organization. Depression And Other Common Mental Disorders: Global Health Estimates. World Health Organization, 2017 http://apps.who.int/iris/bitstream/handle/10665/254610/WHO-MSD-MER-2017.2-eng.pdf?sequence=1.

[2] Papakostas GI (2009). Major depressive disorder: psychosocial impairment and key considerations in functional improvement. Am. J. Manag. Care.

[3] Sullivan PF, Neale MC, Kendler KS (2000). Genetic epidemiology of major depression: review and meta-analysis. Am. J. Psychiatry.

[4] Wray NR (2012). Gottesman II. Using summary data from the danish national registers to estimate heritabilities for schizophrenia, bipolar disorder, and major depressive disorder. Front. Genet..

[5] Lohoff FW (2010). Overview of the genetics of major depressive disorder. Curr. Psychiatry Rep..

[6] Major Depressive Disorder Working Group of the Psychiatric GWAS Consortium. (2013). A mega-analysis of genome-wide association studies for major depressive disorder. Mol. Psychiatry.

[7] Gamazon ER, Zwinderman AH, Cox NJ, Denys D, Derks EM (2019). Multi-tissue transcriptome analyses identify genetic mechanisms underlying neuropsychiatric traits. Nat. Genet..

[8] Gratten J, Wray NR, Keller MC, Visscher PM (2014). Large-scale genomics unveils the genetic architecture of psychiatric disorders. Nat. Neurosci..

[9] Belmaker RH, Agam G (2008). Major depressive disorder. N. Engl. J. Med..

[10] Kessler RC, Chiu WT, Demler O, Merikangas KR, Walters EE (2005). Prevalence, severity, and comorbidity of 12-month DSM-IV disorders in the National Comorbidity Survey Replication. Arch. Gen. Psychiatry.

[11] Albert PR (2015). Why is depression more prevalent in women?. J. Psychiatry Neurosci..

[12] Addis ME (2008). Gender and Depression in Men. Clin. Psychol.: Sci. Pract..

[13] Labonté B (2017). Sex-specific transcriptional signatures in human depression. Nat. Med..

[14] Seney ML (2018). Opposite molecular signatures of depression in men and women. Biol. Psychiatry.

[15] Howard DM (2019). Genome-wide meta-analysis of depression identifies 102 independent variants and highlights the importance of the prefrontal brain regions. Nat. Neurosci..

[16] Hyde CL (2016). Identification of 15 genetic loci associated with risk of major depression in individuals of European descent. Nat. Genet..

[17] Howard DM (2018). Genome-wide association study of depression phenotypes in UK Biobank identifies variants in excitatory synaptic pathways. Nat. Commun..

[18] Wray NR (2018). Genome-wide association analyses identify 44 risk variants and refine the genetic architecture of major depression. Nat. Genet..

[19] de Leeuw CA, Mooij JM, Heskes T, Posthuma D (2015). MAGMA: generalized gene-set analysis of GWAS data. PLoS Comput. Biol..

[20] Ding Y (2015). Molecular and genetic characterization of depression: overlap with other psychiatric disorders and aging. Mol. Neuropsychiatry.

[21] Ramaker RC (2017). Post-mortem molecular profiling of three psychiatric disorders. Genome Med..

[22] Torre D, Lachmann A, Ma’ayan A (2018). BioJupies: automated generation of interactive notebooks for RNA-Seq Data analysis in the cloud. Cell Syst..

[23] Smyth G. K. in *Bioinformatics and Computational Biology Solutions Using R and Bioconductor* (eds Gentleman, R,, Carey, V., Dudoit, S., Irizarry, R. & Huber, W. http://citeseerx.ist.psu.edu/viewdoc/summary?doi=10.1.1.363.443 (Accessed 24 Jan 2020).

[24] Phipson B, Lee S, Majewski IJ, Alexander WS, Smyth GK (2016). Robust hyperparameter estimation protects against hypervariable genes and improves power to detect differential expresSION. Ann. Appl. Stat..

[25] Law CW, Chen Y, Shi W, Smyth GK (2014). voom: Precision weights unlock linear model analysis tools for RNA-seq read counts. Genome Biol..

[26] Hawrylycz M (2015). Canonical genetic signatures of the adult human brain. Nat. Neurosci..

[27] Fisher R. A. 224A: Answer to Question 14 on Combining independent tests of significance. *Am. Statistician* https://digital.library.adelaide.edu.au/dspace/handle/2440/15258 (1948).

[28] Hawrylycz MJ (2012). An anatomically comprehensive atlas of the adult human brain transcriptome. Nature.

[29] Arloth J, Bader DM, Röh S, Altmann A (2015). Re-Annotator: annotation pipeline for microarray probe sequences. PLoS ONE.

[30] Zeisel, A. et al. Molecular architecture of the mouse nervous system. *bioRxiv*10.1101/294918 (2018). 10.1016/j.cell.2018.06.021 PMC6086934 30096314

[31] Ogan Mancarcı B. h*omologene*. Github https://github.com/oganm/homologene (Accessed 11 Mar 2020).

[32] Labaka A, Goñi-Balentziaga O, Lebeña A, Pérez-Tejada J (2018). Biological sex differences in depression: a systematic review. Biol. Res. Nurs..

[33] Doumas M, Smolders C, Brunfaut E, Bouckaert F, Krampe RTH (2012). Dual task performance of working memory and postural control in major depressive disorder. Neuropsychology.

[34] Monteiro, S. et al. Association between depression severity and executive functioning in late-life depression: a systematic review. *Med. Express* **3**. 10.5935/MedicalExpress.2016.06.01 (2016).

[35] Alves MRP (2014). Executive function impairments in patients with depression. CNS Neurol. Disord. Drug Targets.

[36] Brooks BL, Iverson GL, Sherman EMS, Roberge M-C (2010). Identifying cognitive problems in children and adolescents with depression using computerized neuropsychological testing. Appl. Neuropsychol..

[37] Vergara-Lopez C, Lopez-Vergara HI, Colder CR (2013). Executive functioning moderates the relationship between motivation and adolescent depressive symptoms. Pers. Individ Dif..

[38] Ajilchi B, Nejati V (2017). Executive functions in students with depression, anxiety, and stress symptoms. Basic Clin. Neurosci..

[39] Sedeño-Cortés AE, Pavlidis P (2014). Pitfalls in the application of gene-set analysis to genetics studies. Trends Genet..

[40] Crow M, Lim N, Ballouz S, Pavlidis P, Gillis J (2019). Predictability of human differential gene expression. Proc. Natl Acad. Sci. USA.

[41] Pandya M, Altinay M, Malone DA, Anand A (2012). Where in the brain is depression?. Curr. Psychiatry Rep..

[42] Murray EA, Wise SP, Drevets WC (2011). Localization of dysfunction in major depressive disorder: prefrontal cortex and amygdala. Biol. Psychiatry.

[43] Salvadore G (2011). Prefrontal cortical abnormalities in currently depressed versus currently remitted patients with major depressive disorder. Neuroimage.

[44] Schmaal L (2016). Subcortical brain alterations in major depressive disorder: findings from the ENIGMA Major Depressive Disorder Working group. Mol. Psychiatry.

[45] Schmaal L (2017). Cortical abnormalities in adults and adolescents with major depression based on brain scans from 20 cohorts worldwide in the ENIGMA Major Depressive Disorder Working Group. Mol. Psychiatry.

[46] van der Aa HPA, Comijs HC (2015). Penninx BWJH, van Rens GHMB, van Nispen RMA. Major depressive and anxiety disorders in visually impaired older adults. Invest. Ophthalmol. Vis. Sci..

[47] Choi HG, Lee MJ, Lee S-M (2018). Visual impairment and risk of depression: a longitudinal follow-up study using a national sample cohort. Sci. Rep..

[48] Fam J, Rush AJ, Haaland B, Barbier S, Luu C (2013). Visual contrast sensitivity in major depressive disorder. J. Psychosom. Res..

[49] Evans JR, Fletcher AE, Wormald RPL (2007). Depression and anxiety in visually impaired older people. Ophthalmology.

[50] Garcia GA (2017). Profound vision loss impairs psychological well-being in young and middle-aged individuals. Clin. Ophthalmol..

[51] Rovner BW, Ganguli M (1998). Depression and disability associated with impaired vision: the MoVies Project. J. Am. Geriatr. Soc..

[52] Shaqiri A (2018). Sex-related differences in vision are heterogeneous. Sci. Rep..

[53] Peirce JM, Alviña K (2019). The role of inflammation and the gut microbiome in depression and anxiety. J. Neurosci. Res..

[54] Ch’ng C, Kwok W, Rogic S, Pavlidis P (2015). Meta-analysis of gene expression in autism spectrum disorder. Autism Res..

[55] Schizophrenia Working Group of the Psychiatric Genomics Consortium. (2014). Biological insights from 108 schizophrenia-associated genetic loci. Nature.

[56] Jaffe AE (2018). Developmental and genetic regulation of the human cortex transcriptome illuminate schizophrenia pathogenesis. Nat. Neurosci..

[57] Barbu M. C. et al. Epigenetic prediction of major depressive disorder. *Mol. Psychiatry*10.1038/s41380-020-0808-3 (2020). 10.1038/s41380-020-0808-3 PMC8589651 32523041

[58] Clark SL (2020). A methylation study of long-term depression risk. Mol. Psychiatry.

[59] Nagy C (2020). Single-nucleus transcriptomics of the prefrontal cortex in major depressive disorder implicates oligodendrocyte precursor cells and excitatory neurons. Nat. Neurosci..

[60] Kious, B. M., Kondo, D. G. & Renshaw, P. F. Creatine for the treatment of depression. *Biomolecules* **9**, 10.3390/biom9090406 (2019). 10.3390/biom9090406 PMC6769464 31450809

[61] Ramírez O, Jiménez E (2002). Sexual dimorphism in rat cerebrum and cerebellum: different patterns of catalytically active creatine kinase isoenzymes during postnatal development and aging. Int J. Dev. Neurosci..

[62] Uhlén M (2015). Proteomics. Tissue-based map of the human proteome. Science.

[63] Allen PJ, D’Anci KE, Kanarek RB, Renshaw PF (2010). Chronic creatine supplementation alters depression-like behavior in rodents in a sex-dependent manner. Neuropsychopharmacology.

[64] Kondo DG (2016). Creatine target engagement with brain bioenergetics: a dose-ranging phosphorus-31 magnetic resonance spectroscopy study of adolescent females with SSRI-resistant depression. Amino Acids.

[65] Bakian AV, Huber RS, Scholl L, Renshaw PF, Kondo D (2020). Dietary creatine intake and depression risk among U.S. adults. Transl. Psychiatry.

[66] Samaan Z (2015). Obesity genes and risk of major depressive disorder in a multiethnic population: a cross-sectional study. J. Clin. Psychiatry.

[67] Kilpeläinen TO (2011). Genetic variation near IRS1 associates with reduced adiposity and an impaired metabolic profile. Nat. Genet..

[68] DIAbetes Genetics Replication And Meta-analysis (DIAGRAM) Consortium. (2014). Genome-wide trans-ancestry meta-analysis provides insight into the genetic architecture of type 2 diabetes susceptibility. Nat. Genet..

[69] Lu Y (2016). New loci for body fat percentage reveal link between adiposity and cardiometabolic disease risk. Nat. Commun..

[70] Cook NL (2019). CRISPR-Cas9-mediated knockout of SPRY2 in human hepatocytes leads to increased glucose uptake and lipid droplet accumulation. BMC Endocr. Disord..

[71] Li L, Gower BA, Shelton RC, Wu X (2017). Gender-specific relationship between obesity and major depression. Front Endocrinol..

[72] Zhou W (2017). Neddylation E2 UBE2F promotes the survival of lung cancer cells by activating CRL5 to degrade NOXA via the K11 linkage. Clin. Cancer Res..

[73] Li L (2014). Overactivated neddylation pathway as a therapeutic target in lung cancer. J. Natl Cancer Inst..

[74] Yu J. et al. Overactivated neddylation pathway in human hepatocellular carcinoma. *Cancer Med*. 2018. 10.1002/cam4.1578 (2018). 10.1002/cam4.1578 PMC6051160 29846044

[75] Gupta S, Kim S-M, Wang Y, Dinasarapu AR, Subramaniam S (2014). Statistical insights into major human muscular diseases. Hum. Mol. Genet..

[76] Park CK (2019). Neuromyositis: a rare extramuscular manifestation of dermatomyositis. J. Rheum. Dis..

[77] Nguyen TP, Bangert C, Biliciler S, Athar P, Sheikh K (2014). Dermatomyositis-associated sensory neuropathy: a unifying pathogenic hypothesis. J. Clin. Neuromuscul. Dis..

[78] Irie T (2016). Dermatomyositis complicated with asymmetric peripheral neuritis on exacerbation: a case report and literature review. Clin. Exp. Neuroimmunol..

[79] Wang Y (2010). Nerve conduction studies in patients with dermatomyositis or polymyositis. Chin. Med. J..

[80] Nomura M (2010). Adult dermatomyositis with severe polyneuropathy: does neuromyositis exist?. Neurol. Sci..

[81] Vogelgesang SA, Gutierrez J, Klipple GL, Katona IM (1995). Polyneuropathy in juvenile dermatomyositis. J. Rheumatol..

[82] Matsui N (2003). Dermatomyositis with peripheral nervous system involvement: activation of vascular endothelial growth factor (VEGF) and VEGF receptor (VEGFR) in vasculitic lesions. Intern. Med..

[83] Furuichi T (1989). Primary structure and functional expression of the inositol 1,4,5-trisphosphate-binding protein P400. Nature.

[84] Bouby N, Trinh-Trang-Tan MM, Bankir L (1984). Stimulation of tubular reabsorption of magnesium and calcium by antidiuretic hormone in conscious rats. Study in Brattleboro rats with hereditary hypothalamic diabetes insipidus. Pflug. Arch..

[85] Hanouna G., Haymann J.-P., Baud L., Letavernier E. Vasopressin regulates renal calcium excretion in humans. *Physiol. Rep.* **3**, 10.14814/phy2.12562 (2015). 10.14814/phy2.12562 PMC4673622 26620256

[86] Morales-Medina, J. C., Witchey, S. K. & Caldwell, H. K. in *Melatonin, Neuroprotective Agents and Antidepressant Therapy* (eds López-Muñoz, F., Srinivasan, V., de Berardis, D., Álamo, C. & Kato, T. A.) 667–685 (Springer, New Delhi, India, 2016).

[87] Neumann ID, Landgraf R (2012). Balance of brain oxytocin and vasopressin: implications for anxiety, depression, and social behaviors. Trends Neurosci..

[88] Müller MB, Landgraf R, Keck ME (2000). Vasopressin, major depression, and hypothalamic-pituitary-adrenocortical desensitization. Biol. Psychiatry.

[89] Case, K. C., Salsaa, M., Yu, W. & Greenberg, M. L. Regulation of inositol biosynthesis: balancing health and pathophysiology. *Handb. Exp. Pharmacol.*10.1007/164_2018_181 (2018). 10.1007/164_2018_181 30591968

